# Molecular characterisation and taxon assemblage typing of giardiasis in primary school children living close to the shoreline of Lake Albert, Uganda

**DOI:** 10.1016/j.parepi.2018.e00074

**Published:** 2018-11-23

**Authors:** Hajri Al-Shehri, E. James LaCourse, Otto Klimach, Narcis B. Kabatereine, J. Russell Stothard

**Affiliations:** a Department of Parasitology, Liverpool School of Tropical Medicine, Liverpool L3 5QA, UK; b Ministry of Health, Asir District, Kingdom of Saudi Arabia; c Vector Control Division, Ministry of Health, Kampala, Uganda

**Keywords:** *Giardia duodenalis*, Real-time PCR, Assemblage B, *β*-giardin, Wasting

## Abstract

As part of an epidemiological survey for gastrointestinal parasites in school children across five primary schools on the shoreline of Lake Albert, the prevalence of giardiasis was 87.0% (n = 254) as determined by real-time PCR analysis of faecal samples with a genus-specific *Giardia* 18S rDNA probe. Faecal samples were further characterised with taxon assemblage-specific triose phosphate isomerase (TPI) Taqman® probes and by sequence characterisation of the *β*-giardin gene. While less sensitive than the 18S rDNA assay, general prevalence by TPI probes was 52.4%, with prevalence by taxon assemblage of 8.3% (assemblage A), 35.8% (assemblage B) and 8.3% co-infection (A & B assemblages). While assemblage B was dominant across the sample, proportions of assemblages A and B, and co-infections thereof, varied by school and by age of child; mixed infections were particularly common at Runga school (OR = 6.9 [95% CI; 2.5, 19.3]) and in children aged 6 and under (OR = 2.7 [95% CI; 1.0, 7.3]). Infection with assemblage B was associated with underweight children (OR = 2.0 [95% CI; 1.0, 3.9]). The presence of each assemblage was also confirmed by sequence analysis of the *β*-giardin gene finding sub-assemblage AII and further genetic diversity within assemblage B. To better explore the local epidemiology of giardiasis and its impact on child health, additional sampling of school children with assemblage typing would be worthwhile.

## 1 Introduction

The binucleate flagellated protozoan *Giardia duodenalis* (syn. *G. lamblia, G. intestinalis*) is a common gastrointestinal parasite able to infect a variety of mammals (Adam, 2001; Helmy et al., 2014). Where sanitation and hygiene are poor, these parasites can cause acute and/or chronic giardiasis across all ages (Wegayehu et al., 2016; Muhsen and Levine, 2012; Rogawski et al., 2017; Tellevik et al., 2015). While levels of endemicity of giardiasis may vary across the world, it can be common in children living within low and middle income countries (Laishram et al., 2012; Muhsen and Levine, 2012); for example, in Uganda giardiasis can be particularly rife (Al-Shehri et al., 2016; Fuhrimann et al., 2016), but its effect on child health is not fully appreciated but in Rwanda nearby, the very high prevalence of *G. duodenalis* in children aged 5 and under, was associated with being underweight (Ignatius et al., 2012).

There are eight distinct groups or taxonomic assemblages (A to H) within *Giardia* currently recognised (Sprong et al., 2009; Almeida et al., 2010; Takumi et al., 2012). Assemblages A and B are typically held most responsible for human infections, with the latter assemblage associated with zoonotic transmission (Almeida et al., 2010; Feng and Xiao, 2011; Vanni et al., 2012; Asher et al., 2014; Thompson and Ash, 2016); each assemblage can be further divided into sub-assemblages, e.g. A: AI, AII & AIII and B: BIII and BIV on the basis of sequence variation within molecular markers e.g. glutamate dehydrogenase (GDH), β-giardin, small subunit ribosomal DNA (18S rDNA), and triose phosphate isomerase (TPI) (Durigan et al., 2014; Karim et al., 2015; Minetti et al., 2015). Despite efforts to investigate specific assemblages with disease symptoms and severity, there is no absolute association to date (Sprong et al., 2009; Thompson and Ash, 2016).

In Uganda, general investigations on the epidemiology of giardiasis are increasing (Nizeyi et al., 1999; Graczyk et al., 2002; Nizeyi et al., 2002; Johnston et al., 2010), although only a single study has employed molecular methods of characterisation (Ankarklev et al., 2012). Ankarklev et al. (2012) investigated associations between taxon assemblages and *Helicobacter pylori* infection in apparently healthy children aged 0–12 living in Kampala, the capital. Assemblage B was found dominant and a risk factor for *H. pylori* infection (Ankarklev et al., 2012) and like in other parts of the world, assemblage B was more associated with symptomatic infections (Pelayo et al., 2008; Puebla et al., 2014).

To shed light on the taxonomic assemblages of *Giardia* within school children living on the shoreline of Lake Albert, we undertook a molecular characterisation of previously characterised stool samples as reported by Al-Shehri et al. (Al-Shehri et al., 2016). Faecal samples were further characterised with assemblage-specific TaqMan® TPI probes and the presence of each taxon assemblage confirmed by sequence analysis of the β-giardin gene. Associations between taxon assemblage and collected epidemiological data were explored.

## 2 Materials and methods

### 2.1 Faecal material and epidemiological information

Faecal samples were available for further molecular analysis (see below) that were initially collected within the epidemiological survey of 254 school children from five primary schools (Bugoigo, Runga, Walakuba, Biiso and Busingiro) as reported by Al-Shehri et al. (Al-Shehri et al., 2016). Each sampled child underwent an epidemiological questionnaire and clinical examination; data on socio-demographical aspects and standard biometry were recorded (height with a clinical stadiometer, model 214; SECA, Hanover, MD and weight by weighing scales with a model 803; SECA, Hanover; MD). Heights and weights were used to assess stunting, height-for-age *Z*-score (HAZ), and wasting, weight-for-age Z-score (WAZ). Children were defined as stunted if their height-for-age Z score was −2 ≤ SD and underweight if their weight-for-age Z score was −2 ≤ SD (WHO, 2007). Finger-prick blood was collected from each child and tested for haemoglobin levels by HemoCue® portable haemoglobin photometer (HemoCue, CA 92630, USA). Children were considered anaemic if haemoglobin levels were below 115 g/L (WHO, 2011).

During the surveys, all sampled stools were tested for faecal occult blood (Mission Test, Acon Laboratories, San Diego, CA, USA) but owing to a limited supply of rapid diagnostic tests (RDTs), only stools collected from Bugiogo and Runga were tested in-field with Quik-Chek RDTs for giardiasis and cryptosporidiosis (GIADIA/CRYPTOSPORIDIUM Quik-Chek, Alere, Galway, Ireland). Stools were then stored in absolute ethanol for later DNA analysis.

### 2.2 Molecular profiling of *G. duodenalis* assemblages

After transfer to the UK and each faecal sample was spiked with Phocine Herpes Virus to act as an internal control for genomic DNA extraction and amplification performance of later real-time PCR assays. Genomic DNA was extracted, and detection of *Giardia* 18S rDNA was performed using TaqMan® assay following primers, probes and protocols of Verweij et al. (Verweij et al., 2004). These extractions were again retested with a duplex real-time PCR assay with assemblage-specific A and B probes using the TPI locus (Elwin et al., 2014). The real-time PCR analysis of faecal extractions from each school was completed in separate PCR plates that each contained negative and positive controls; a negative control (without genomic DNA template) of extraction elution buffer (10 mM Tris-HCl [pH 8], 1 mM EDTA) and a positive control (with reference genomic *Giardia* DNA template) from a heavily infected individual excreting approximately 1000 cysts per gram of faeces as estimated by microscopy. As a further quality control, reamplification of 10% of samples was undertaken to assess assemblage assay reliability. Assays were performed in a Chromo-4 with Opticon monitor™ version 3.1. (Bio-Rad, UK). The infection was determined according to C_*t*_ values; for the 18S rDNA TaqMan® assay no-infection was C_*t*_ ≥  40 and positive infection C_*t*_ ≤ 39 while for assemblages-specific probes was C_*t*_ ≤ 45.

To further confirm assemblage A and B, the *β*-giardin gene was amplified from samples from six children using nested PCR following protocols of Minetti et al. (Minetti et al., 2015). PCR products were purified using the QIAquick® PCR purification kit (QIAGEN Ltd.) and were sequenced in both directions by Sanger sequencing. Nucleotide sequences and chromatograms were analysed and edited using Geneious software (Vejlsøvej55, 8600 Silkeborg, Denmark). Sequences from this study were aligned with each other and reference sequences downloaded from GenBank (listed below). The assemblages and sub-assemblages at each locus were identified by BLAST searches against the following reference sequences: *β*-giardin (accession nos. X14185.1–**AI**, AY072723.1–**AII**, DQ650649.1–**AIII**, AY072726.1–**BIII**, AY072725.1–**BIV**).

### 2.3 Statistical analyses

Statistical analysis was performed using Minitab Ltd.® (Brandon Court, Unit E1-E2 Coventry CV3 2TE UK). Binary logistic regression tests were performed to compare data from each school and as well as risk variables as an independent indicator to assess any associations with specific assemblages.

## 3 Results

Out of the 254 samples examined, 221 tested positive (87.0%) by targeting *Giardia* 18S rDNA assay while 133 (52.3%) tested positive with TPI assemblage-specific probes. Across Bugoigo and Runga schools, the prevalence of giardiasis by Quik-Chek RDT was 41.6%. Of the 133-tested positive by TPI probes, 21 samples were positive for assemblage A (15.8%) only, 91 positives for assemblage B (68.4%) only and 21 positives for both assemblage A and B (15.8%), mixed assemblage infections.

**Fig. 1:**
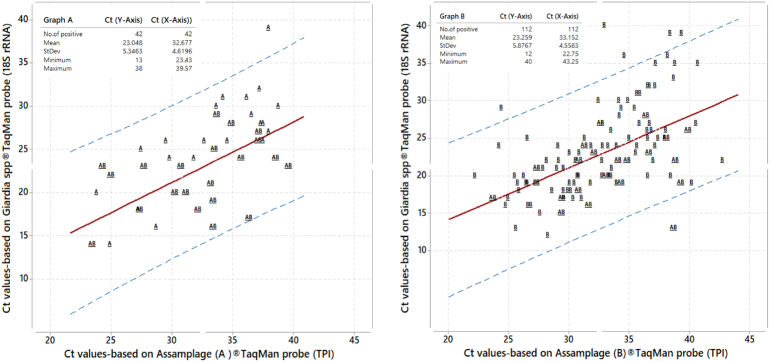
Bivariate plot of Ct values obtained for each sample using *Giardia* TaqMan® 18S rDNA versus Ct value of assemblage-specific TaqMan® TPI probe with dashed lines showing the 95% prediction interval. Fig. 1A. Using assemblage A probe; Fig. 1B Using assemblage B probe.

Across these samples assemblage, A was less common than assemblage B, an approximate ratio of 1: 2.7, with assemblage B dominant. To ascertain if there was any amplification bias in assemblage detection, Fig. 1A shows a bivariate plot of Ct values for *Giardia* 18S rDNA TaqMan® probe and the corresponding Ct value of assemblage A TPI TaqMan® probe (18S rDNA = 0.203 + 0.6991 TPI, with R-squared 34.91% (*P* < 0.005), and positive correlation (r = 0.60)); Fig. 1B shows bivariate plot for assemblage B (18S rDNA = 0.228 + 0.6947 TPI, with R-squared 28.39% (*P* < 0.005), and positive correlation (r = 0.54)). The performance of each TaqMan® assay appeared equivalent. Of note, however, is that mixed assemblage infections appear more common at Runga school where the local prevalence of assemblage A was also much higher.

Table 2 shows epidemiological associations cross-tabulated against available assemblage information. Most notable is the association of mixed assemblages in younger children (OR = 2.7 [95% CI; 1.0, 7.3]) that assemblage B was associated with the presence of faecal occult blood (OR = 2.2 [95% CI; 1.0, 5.2]). It appeared that there was also a significant association of infection with assemblage B and children of lower weight-for-age, i.e. wasting (OR = 2.0 [95% CI; 1.0, 3.9]).

Table 3 details the point mutations with the six representative samples for the *β*-giardin gene, finding an exact match with sub-assemblage AII and no sequence within the three sample inspected. By contrast, each of the three samples for assemblage B was different and did not match either BIII and BIV precisely. The sequence from Sample 102 is particularly notable as there appeared to be allelic variation within the TPI gene as evidenced by split-peak chromatograms of A/G or T/C at three locations present within this region (see Annex Supplemental Fig. 1).

## 4 Discussion

The high prevalence of giardiasis reported here by real-time PCR with the 18S rDNA probe analysis (87.0%) demonstrates that children living on the shoreline of Lake Albert are at very high risk of both acute and more likely, chronic infections. The high burden of giardiasis was also corroborated in field by the Quik-Chek RDT at Runga and Bugoigo schools confirming that some 41.6% of children were patently shedding copious amounts of *Giardia* cysts within their stools. It is unsurprising perhaps that the levels are so high since this lakeshore environment has very poor local sanitation and water hygiene, as well as being hyperendemic for intestinal schistosomiasis, an another waterborne disease (Al-Shehri et al., 2016). Nonetheless the prevalence of giardiasis here is much elevated in comparison to other parts of the world (Thompson and Smith, 2001), although in Rwanda over 60% of rural children have been shown to be infected with *Giardia* by molecular typing methods (Ignatius et al., 2014). More broadly, the diagnostic sensitivity of real-time PCR methods is known to be superior to alternative diagnostic methods, often revealing giardiasis to be more pervasive (Gotfred-Rasmussen et al., 2016), and also creates opportunities for investigations of (sub)assemblage transmission dynamics (Thompson and Ash, 2016).

Given the multi-copy nature of the 18S rDNA against the lower copy number of TPI, the diagnostic sensitivity of TPI probes is lower, such that just under a half of the infected cases detected by 18S rDNA were missed. It has been stated previously that the detection limit of *Giardia* 18S rDNA probe assay is approaching 10 pg DNA/μL (Jaros et al., 2011), presumably that of TPI assay is much higher (Elwin et al., 2014) such that assemblage typing of ‘light’ intensity infections is not always possible. A similar level of diagnostic discordance has been observed elsewhere (Ignatius et al., 2014) which hopefully does not lead to a systematic bias in general reporting of each assemblage, as evidenced by Ct values in Fig. 1, but rather that typing parasites with assemblage-specific primers is not possible when shedding cysts are too few in number.

Nonetheless, in this sample assemblage B dominates upon comparison to assemblage A. Notably this 1:2.7 ratio varied by school with Runga having a greater proportion of assemblage A, as well as co-infection with assemblage B thereof, see Table 1, and more broadly, there appeared to be some interesting epidemiological associations by assemblage, see Table 2. Although there was no association with gender, younger children appeared to harbour a greater proportion of mixed assemblage infections than older counterparts (OR = 2.7 [95% CI; 1.0, 7.3]). There was also an indication that faecal occult blood was associated with assemblage B (OR = 2.2 [95% CI; 1.0, 5.2]) and in children being underweight (OR = 2.0 [95% CI; 1.0, 3.9]). These findings add to the general debate on the health consequences of giardiasis with particular emphasis on assemblage B, which also appears more genetically heterogeneous that assemblage A here (Thompson and Ash, 2016).

**Table 1:** Prevalence (%) of *G. duodenalis* and assemblages across all five schools by real-time PCR; the odds ratio of assemblages A, B or A/B by school compared against the total given (with 95% confidence limits).

School	Giardia TaqMan® 18S rDNA probe		Assemblage (A & B) TaqMan® TPI probe				
	Number of positives % (x/y)	95% CL	Number of positives % (x/y)	95% CL	A % (x/y) OR [95% CI]	B % (x/y) OR [95% CI]	AB % (x/y) OR [95% CI]
Bugoigo	94.5% (52/55)	[85.8–98.6]	56.3% (31/55)	[43.1–69.0]	5.4% (3/55) **0.6 [0.2, 2.5]**	43.6% (24/55) **1.4 [0.8, 2.8]**	7.2% (4/55) **0.9 [0.3, 3.1]**
Runga	94.1% (48/51)	[84.8–98.5]	72.5% (37/51)	[59.2–83.4]	15.6% (8/51) **4.7 [1.7, 13.3]**	37.2% (19/51) **2.0 [1.0, 4.3]**	19.6% (10/51) **6.9 [2.5, 19.3]**
Walukuba	88.0% (44/50)	[76.7–95.0]	40.0% (20/50)	[27.2–54.0]	2.0% (1/50) **0.1 [0.0, 1.2]**	32.0% (16/50) **0.6 [0.3, 1.8]**	6.0% (3/50) **0.5 [0.1, 1.8]**
Biiso	84.0% (42/50)	[71.9–92.3]	54.0% (27/50)	[40.2–67.4]	14.0% (7/50) **2.1 [0.8, 5.9]**	36.0% (18/50) **1.0 [0.5, 2.1]**	4.0% (2/50) **0.4 [0.1, 2.1]**
Busingiro	72.9% (35/48)	[59.1–84.0]	37.5% (18/48)	[24.7–51.8]	4.1% (2/48) **0.3 [0.1, 1.5]**	29.1% (14/48) **0.5 [0.3, 1.1]**	4.1% (2/48) **0.3 [0.1, 1.5]**
All	87.0% (221/254)	[82.4–90.7]	52.4% (133/254)	[46.2–58.5]	8.3% (21/254) ——————	35.8% (91/254) ——————	8.3% (21/254) ——————

**Table 2:** Analysis of potential epidemiological associations by binary logistic regression with *Giardia* assemblages A, B or A/B co-infection.

Epidemiological factors	Assemblage (A, B & AB)® TaqMan probe (TPI)					
	Infected with A	OR [95 CL]	Infected with B	OR [95 CL]	Infected with AB	OR [95 CL]
Gender						
Male	10	1.0 [0.4, 2.7]	44	1.0 [0.6, 1.9]	12	1.5 [0.6, 3.9]
Female	11	0.9 [0.4, 2.4]	47	0.9 [0.5, 1.6]	9	0.6 [0.3, 1.6]
Age group						
5 to 6	11	1.5 [0.6, 3.8]	38	0.9 [0.6, 1.7]	14	2.7 [1.0, 7.3]
7 to 8	9	0.9 [0.4, 2.5]	34	0.7 [0.5, 1.4]	6	0.5 [0.2, 1.5]
9 to 10	1	0.2 [0.0, 2.3]	19	1.5 [0.7, 3.1]	1	0.2 [0.0, 2.3]
Faecal occult blood (FOB)						
Negative	18	0.6 [0.2, 2.4]	74	0.4 [0.2, 1.0]	17	0.4 [0.1, 1.5]
Positive	3	1.6 [0.4, 6.6]	17	2.2 [1.0, 5.2]	4	2.3 [0.7, 8.2]
Height-for-age Z score, mean						
−2 > SD height-for-age Z score	17	1.3 [0.4, 4.3]	63	0.7 [0.4, 1.3]	14	0.6 [0.2, 1.7]
−2 ≤ SD height-for-age Z score	4	0.7 [0.2, 2.4]	28	1.4 [0.8, 2.6]	7	1.5 [0.6, 4.3]
Weight-for-age Z score, mean						
−2 > SD weight-for-age Z score	20	3.9 [0.5,31.2]	65	0.4 [0.3, 1.0]	16	0.6 [0.2, 1.9]
−2 ≤ SD weight-for-age Z score	1	0.2 [0.0, 2.0]	26	2.0 [1.0, 3.9]	5	1.5 [0.5, 4.8]
Anaemia (<115 Hbg/L)						
Negative	9	0.4 [0.1, 1.4]	46	0.5 [0.3, 1.2]	9	0.3 [0.1, 1.0]
Positive	6	2.3 [0.7, 7.5]	23	1.7 [0.8, 3.7]	8	3.1 [1.1, 9.4]
Not determined	6	–	22	–	4	–

It is an interesting observation that of the six samples subjected to sequence analysis of *β*-giardin, the three samples selected from assemblage A were identical and could be further unequivocally assigned to sub-lineage AII, which has been reported in other studies (Cacciò and Ryan, 2008; Plutzer et al., 2010; Cacciò and Sprong, 2010; Ryan and Cacciò, 2013; Beck et al., 2012; Zhang et al., 2012). By contrast, of the three samples selected from assemblage B, there were each different, see Table 3, and none matched exactly either BIII or BIV sub-assemblages. Most notable are the point mutations at positions 176, 188 and 314, where split-peak chromatograms were observed (see Annex). This is indicative of mixed amplicon templates inferring putative allelic variation within the TPI locus. The genomic complexity of *Giardia* is complex, being binucleate and sometimes aneuploid (Aguiar et al., 2016) which might infer sample 102 was either a mixed co-infection of two independent B lineages or contains a single infection lineage with an unusual genomic TPI variant. Nonetheless, there is greater diversity within assemblage B and with further genetic profiling would reveal additional variants which might point towards currently unknown heterogeneities in local transmission cycles. For example, there is numerous livestock e.g. cattle and goats, that regularly enter into the lake and while drinking openly defecate into the water which may add to raised zoonotic potential in such domestic water directly drawn from the lake.

**Table 3:** Single nucleotide polymorphisms within β-giardin of *Giardia duodenalis.*

Assemblage	Isolate/Genbank number	Nucleotide position								
A isolates	Beta-giardin (bg)	**284**	**383**	**407**	**473**	**491**	**563**	**593**	**596**	**611**
**AI**	X14185.1	C	T	T	T	A	G	T	C	A
**AII**	AY072723.1	C	T	T	T	A	G	T	T	A
**AIII**	DQ650649.1	T	C	C	C	G	A	C	C	G
	Sample 9	C	T	T	T	A	G	T	T	A
	Sample 22	C	T	T	T	A	G	T	T	A
	Sample 103	C	T	T	T	A	G	T	T	A
B isolates		**170**	**176**	**188**	**233**	**287**	**314**	**317**	**398**	
**BIII**	AY072726.1	C	A	A	G	C	C	C	C	
**BIV**	AY072725.1	T	A	A	A	T	T	T	T	
	Sample 24	C	A	A	A	C	T	T	C	
	Sample 104	C	A	A	A	C	C	T	C	
	Sample 102	C	A/G	A/G	A	C	T/C	T	C	

To conclude, additional sampling of school children would be worthwhile if putative associations between assemblage B and detrimental health outcomes reported here are to be fully verified statistically. Furthermore to better monitor local transmission cycles of *Giardia*, we encourage future studies that track each assemblage within local livestock and undertake environmental sampling of lake water where domestic water is drawn.

The following is the supplementary data related to this article.

Supplemental Fig. 1 DNA chromatograms illustrative of genetic variation at each variable position. Supplemental Fig. 1
