# Supplementary figures and images for: Molecular characterisation and taxon assemblage typing of giardiasis in primary school children living close to the shoreline of Lake Albert, Uganda
Source: Parasite Epidemiol Control. 2018 Nov 23;4:e00074. doi: 10.1016/j.parepi.2018.e00074 (PMC6324016; doi:10.1016/j.parepi.2018.e00074)

## Slide 1
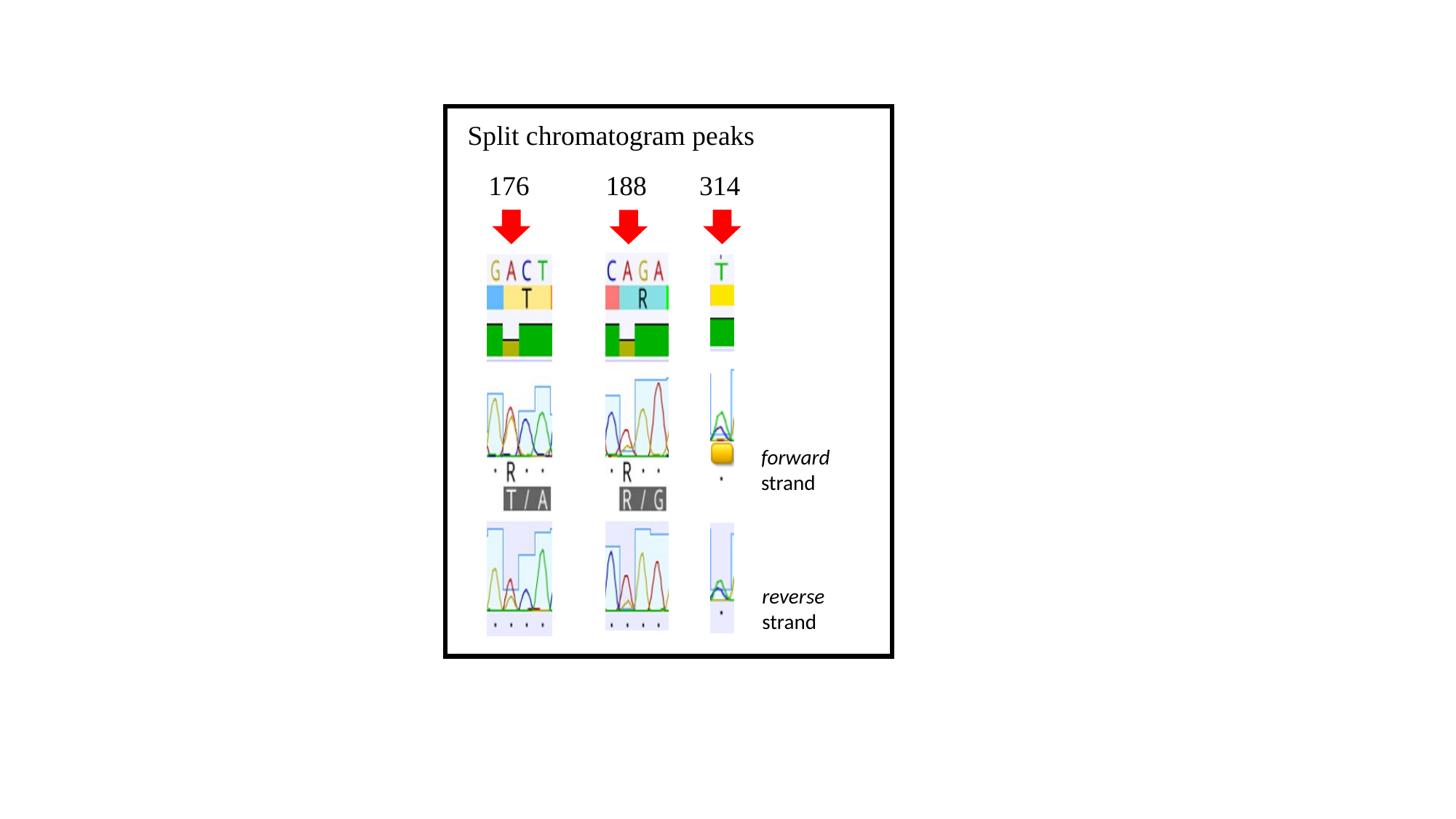

Split chromatogram peaks
176
188
314
forward
strand
reverse
strand

Supplement: Supplemental Fig. 1 — DNA chromatograms illustrative of genetic variation at each variable position. [file mmc1.pptx]
